# Cranial shape variation in domestication: A pilot study on the case of rabbits

**DOI:** 10.1002/jez.b.23171

**Published:** 2022-08-07

**Authors:** Madeleine Geiger, Marcelo R. Sánchez‐Villagra, Emma Sherratt

**Affiliations:** ^1^ Paleontological Institute and Museum University of Zurich Zurich Switzerland; ^2^ Naturmuseum St.Gallen St.Gallen Switzerland; ^3^ SWILD, Urban Ecology & Wildlife Research Zurich Switzerland; ^4^ School of Biological Sciences University of Adelaide Adelaide SA Australia

**Keywords:** allometry, cranium, modularity, *Oryctolagus cuniculus*

## Abstract

Domestication leads to phenotypic characteristics that have been described to be similar across species. However, this “domestication syndrome” has been subject to debate, related to a lack of evidence for certain characteristics in many species. Here we review diverse literature and provide new data on cranial shape changes due to domestication in the European rabbit (*Oryctolagus cuniculus*) as a preliminary case study, thus contributing novel evidence to the debate. We quantified cranial shape of 30 wild and domestic rabbits using micro‐computed tomography scans and three‐dimensional geometric morphometrics. The goal was to test (1) if the domesticates exhibit shorter and broader snouts, smaller teeth, and smaller braincases than their wild counterparts; (2) to what extent allometric scaling is responsible for cranial shape variation; (3) if there is evidence for more variation in the neural crest‐derived parts of the cranium compared with those derived of the mesoderm, in accordance with the “neural crest hypothesis.” Our own data are consistent with older literature records, suggesting that although there is evidence for some cranial characteristics of the “domestication syndrome” in rabbits, facial length is not reduced. In accordance with the “neural crest hypothesis,” we found more shape variation in neural crest versus mesoderm‐derived parts of the cranium. Within the domestic group, allometric scaling relationships of the snout, the braincase, and the teeth shed new light on ubiquitous patterns among related taxa. This study—albeit preliminary due to the limited sample size—adds to the growing evidence concerning nonuniform patterns associated with domestication.

## Research highlights

‐ Evidence for some, but not all cranial characteristics of the “domestication syndrome” in rabbits
‐ Evidence for the “neural crest hypothesis” in rabbits
‐ Results illustrate the non‐uniform pattern of morphological diversification in domestication

## Graphical abstract

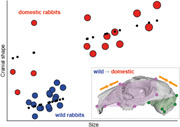

## 1 INTRODUCTION

It is widely acknowledged that the domestication process results in large phenotypic diversity and it is also emphasized how this occurs in historically recent times and thus at a much higher rate than evolution in the wild (e.g., Clutton‐Brock, 1999; Herre & Röhrs, 1990; Sánchez‐Villagra, 2022). Quantification of the phenotypic patterns of domesticated species reveals the extent and mode of such morphological diversification and is the basis of any exploration of evolvability in the context of initial selection on tameness, or the intense selection in breed formation, two extremes of a continuum of the process of domestication. The phenotypic patterns then reveal what changes and the rate at which it does and comparisons across species can then reveal commonalities and differences that generate hypotheses of mechanisms behind them. The skull has been a preferred and rich subject of investigation (e.g., Balcarcel, Sánchez‐Villagra, et al., 2021; Drake & Klingenberg, 2010; Geiger et al., 2017; Heck et al., 2018; Young et al., 2017), as it is complex and correlated with diverse kinds of sensory, developmental and phylogenetic variables.

The idea that there are common patterns of variation across mammalian species has been referred to as “domestication syndrome” (Darwin, 1875; Lord et al., 2020 for a historical overview). This set of phenotypic alterations includes—among other characteristics—cranial shape changes, notably a shortening of the facial part of the skull and a decrease in tooth size, as well as a decrease in brain size (e.g., Herre & Röhrs, 1990 for an overview). The underlying mechanisms of these changes are currently under debate, with the disputed “neural crest hypothesis,” suggesting that selection for tameness leads to mild neural crest cell deficiencies during development, resulting—as a byproduct—in the typical characteristics of the “domestication syndrome” (Kistner et al., 2021; Wilkins et al., 2014; Wilson et al., 2021; for an overview). Recently, it has been pointed out that there are few, if any, universal traits of the “domestication syndrome” across domesticated mammalian species on the nonbreed level (Lord et al., 2020; Sánchez‐Villagra et al., 2017). Thus, traits of the “domestication syndrome” are recorded in many species only for certain, highly derived modern breeds (Lord et al., 2020; but see Zeder, 2020).

There appears to be particularly little evidence for a universal “domestication syndrome” in craniodental traits, including a shortened jaw, a wider face, and smaller and more crowded teeth (Lord et al., 2020; Sánchez‐Villagra et al., 2017). One of the species that has been highlighted in this regard is the domestic rabbit (*Oryctolagus cuniculus*), in which evidence for the presence of craniodental changes as predicted by the “domestication syndrome” hypothesis was reported to be mostly unclear or altogether lacking (Lord et al., 2020). In fact, a couple of works have examined and illustrated cranial shape variation due to domestication and breed formation in rabbits (Darwin, 1875; Hückinghaus, 1965a, b; Klatt, 1913; see also Böhmer & Böhmer, 2017; Fiorello & German, 1997). However, some of these papers are rather dated in the methods used and some are in German and not readily accessible. In the current study, we thus review those older works to make their results and implications more widely available and integrate the findings with the newer studies. Further, we provide new data on craniodental variation in domestic and wild rabbits using three‐dimensional (3D) geometric morphometrics. We hope that this study will not only contribute to our understanding of rabbit domestication, but that it will also add to the ongoing discussions concerning the phenotypic patterns and the diversity of traits that arise in the domestication process (Sánchez‐Villagra, 2022).

The domestication process and subsequent geographical expansion of the European rabbit (*O. cuniculus*, hereafter referred to as “rabbit”) is unique to Western Europe (Carneiro et al., 2011; Ferrand, 2008; Somerville & Sugiyama, 2021). Fossil evidence points to an origin of rabbits on the Iberian Peninsula, where the species was also confined in the early Holocene (Lopez‐Martinez, 2008). Although the timing of onset of the domestication process in rabbits remains elusive, molecular data suggest that the species expanded its range from France across Europe from the Middle Ages onwards, probably facilitated by human‐induced habitat changes, as well as by direct human transfer in the course of domestication (Ferrand, 2008 and references therein; Carneiro et al., 2011; Irving‐Pease et al., 2018). In modern times, the species inhabits all continents (except Antarctica) (Smith & Boyer, 2007) and is used for meat, fur, wool, and as pet and laboratory animal (Robinson, 1984; Weisbroth et al., 1974).

As body size and shape are inherently linked (Gould, 1966), craniodental variation must be investigated in the context of allometric scaling patterns. Rabbits are among the domesticates with the most marked body size changes due to domestication and breed formation, with some domestic breeds reaching multiple times the size and mass of the wild form (Bökönyi, 1974; Hückinghaus, 1965a,b; Robinson, 1984). This is not only remarkable concerning scale, but the pattern of a general body size increase in rabbit domestication also contrasts with the generally observed body size decrease in domestication of most other species (Hückinghaus, 1965a, 1965b; Tchernov & Horwitz, 1991).

In this study, we first quantify craniodental shape variation in rabbits and examine the results in regard to features of the “domestication syndrome.” Specifically, we test whether facial length as well as brain and tooth size are reduced, and whether facial width is increased in the domestic sample. Second, we test whether cranial shape variation observed between wild and domestic rabbits is attributable to size differences between the two groups. We also scrutinize allometric scaling relationships among domestic rabbits to shed further light on underlying patterns. Third, we test whether neural crest‐derived parts of the skull are more variable compared with the mesoderm‐derived parts in line with the “neural crest hypothesis” of the “domestication syndrome.”

## 2 RESULTS

**Figure 1:**
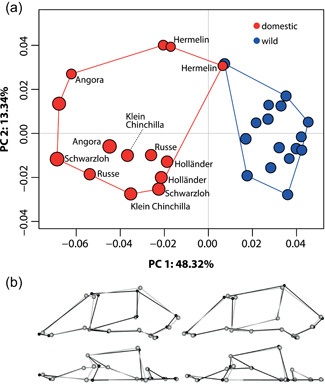
Results of allometry‐included principal component analysis (PCA) comparing cranial shape in domestic and wild rabbits. In the cranial shape morphospace (a) colors denote the wild (blue) and the domestic (red) groups, and plotted points are scaled to centroid size. Names of domestic breeds (where known) are given. Convex hulls outline the portion of morphospace occupied by the wild and the domestic group, respectively. Wireframes (b) show the skull shapes of the most extreme specimens (in black) relative to the consensus shape (in gray), representing the most negative PC1 scores (left) and most positive PC1 scores (right). Depicted are wireframes in lateral (top) and dorsal (bottom) views (see also Figure 2).

Principal component analysis (PCA) of allometry‐included landmark data resulted in the first two principal components (PCs) explaining 61.6% of the variation in the data, with PC1 explaining 48.3% and PC2 13.3% (Figure 1). The wild and domesticated groups were found to be separated along PC1, with the wild group being nested towards the positive and the domestic group towards the negative values, respectively (Figure 1a). The wild and domestic groups shared shape variation described by PC2 (Figure 1a). A shift from positive to negative PC1 values, that is, from wild to domestic, is indicative of a shift to a relatively longer snout, a dorsoventrally shallower braincase, a mediolaterally narrower zygomatic arch, a shorter cheek tooth row, and less pronounced klinorhynchy (i.e., a larger angle between the plane of the cranial base and the palate) (Figure 1b). Further, there is a very slight indication of a broader muzzle in domestic rabbits compared to the wild ones (Figure 1b). Procrustes analysis of variance (ANOVA) revealed significant differences in shape between the two groups (F_(1,28)_ = 4.04, *p* < 0.001) and morphological disparity analysis revealed that domestic specimens had significantly greater shape variation than wild ones (Procrustes variance, domestic = 0.00352; wild = 0.00211; *p* = 0.008).

**Figure 2:**
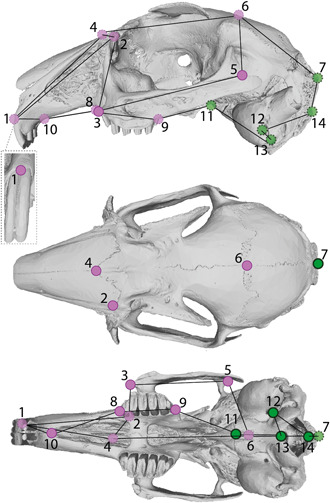
Landmarks and wireframes used in this study. Landmarks are depicted on a surface reconstruction of a wild rabbit (I.f.H. 5188, mirrored) in lateral (top), dorsal (middle), and ventral (bottom) views, and correspond to numbers in Table 1. The gray dashed inlet box depicts landmark No. 1 on the anterior side of the skull. Colors denote the embryonic origin of the bone tissue on which the landmarks are set, following Mishina and Snider (2014): purple, neural crest origin; green, mesoderm origin (see also Table 1). Light shading of points with a dashed outline indicates landmarks that cannot be depicted properly in the respective aspect, but are shown to illustrate the wireframe (black lines connecting the landmarks).

**Figure 3:**
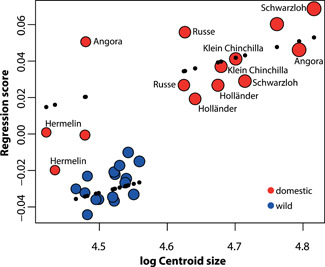
Multivariate regression of cranial shape against log‐transformed centroid size. The regression score represents shape variation attributed to size variation. Colors denote the wild (blue) and the domestic (red) groups, and plotted points are scaled to centroid size. Black dots represent the predicted values of the analysis of covariance, demonstrating the two groups have different allometric trajectories.

Multivariate regression of the shape score against the log centroid size showed that the association between the two is strong (size explains 26.2% of the shape variation) and significant (*p* < 0.001) (Figure 3). However, it is also apparent that the groups do not follow the same allometric trajectory; the two groups have significantly different *y* intercepts (F_(1,28)_ = −4.15, *p* < 0.001), but do not differ in the slope (interaction of size:form F_(1,28)_ = −1.05, *p* = 0.843) (Figure 3).

**Figure 4:**
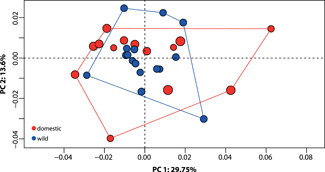
Results of allometry‐adjusted principal component analysis (PCA) comparing cranial shape in domestic and wild rabbits. Colors denote the wild (blue) and the domestic (red) groups, and plotted points are scaled to centroid size. The morphological disparity of domesticates is significantly higher than the wild, as demonstrated by the convex hulls outlining the portion of morphospace occupied by the two groups.

Given the difference in regression slopes, residuals representing allometry‐adjusted landmark data were extracted from the analysis of covariance (ANCOVA), and resulted in the first two PCs explaining 43.35% of the variation in the data, with PC1 explaining 29.8% and PC2 13.6% (Figure 4). Unlike the allometry‐included data (Figure 1a), both groups overlapped in distribution. This indicates there is a common allometric component shared between the two groups, that is offset with size, and when removed the two groups share many shape traits. However, morphological disparity analysis revealed that domestic specimens still had significantly greater shape variation than wild ones (Procrustes variance, domestic = 0.00174; wild = 0.00112; *p* = 0.04).

**Figure 5:**
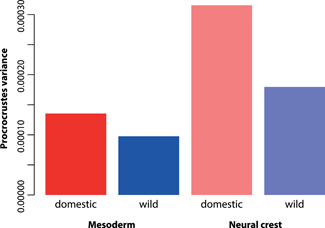
Comparison of Procrustes variances of the mesoderm (solid bars) and the neural crest (transparent)‐derived parts of the skull in wild and domestic rabbits (see also Figure 2). Permutation tests showed that the shape of both modules is by trend more variable in the domestic group compared to the wild one, and that the neural crest is significantly different between wild and domestic rabbits.

Comparison of the Procrustes variance of the mesoderm portion of the cranium in the wild and the domestic sample revealed the domestic was slightly higher but the two were not significantly different (*p* = 0.094) (Figure 5). Concerning the neural crest portion of the cranium, the Procrustes variance was significantly different between the wild and the domestic groups (*p* = 0.008), with domestic having almost twice the amount of variance (Figure 5).

Evaluation of cranial indices from extracted linear cranial dimensions corrected for body size showed that relative facial length was not different in the domestic and the wild group (Wilcoxon–Mann–Whitney *W* = 84, *p* = 0.2572). However, we found that compared with the wild group, the domestic group had a significantly shorter relative tooth row length (*W* = 32, *p* = 0.00005), braincase height (*W* = 27, *p* = 0.0002), braincase length (*W* = 28, *p* = 0.0002), and braincase width (*W* = 24, *p* < 0.001).

Within the domestic sample, facial length scaled isometrically (Slope *b* = 1.14, 95% confidence interval [CI] = 0.82–1.46), braincase height with negative allometry (*b* = 0.62, 95% CI = 0.43–0.81), braincase length with negative allometry (*b* = 0.62, 95% CI = 0.38–0.85), braincase width with negative allometry (*b* = 0.41, 95% CI = 0.07–0.75), and tooth row length with negative allometry (*b* = 0.63, 95% CI = 0.37–0.88) (Supporting Information: Figure S1). In other words, although facial length increased proportionally with body size in domestic rabbits, tooth row length and braincase dimensions were disproportionally large in small domestic varieties—or small in large ones.

## 3 DISCUSSION

Although our study is limited in terms of sample size and coverage of the breadth of wild populations and domestic (e.g., no representatives of giant breeds were included), we find overall patterns of phenotypic change in rabbits are mostly congruent with predicted traits of domestication—as also documented in our synopsis of older works on the subject. Our data then serve to discuss the variation among rabbit breeds in traits deemed as characteristic of the “domestication syndrome” (Lord et al., 2020; Wilkins et al., 2014), so far barely recorded for the species.

### 3.1 Cranial shape changes in rabbit domestication

First, we quantified cranial shape variation comparing wild with domestic rabbits and examine whether such variation is mainly attributable to size differences between the two groups. In previous works, domestic rabbits were found to exhibit relatively longer facial bones and a deeper (higher) skull, with the braincase being less rounded compared with wild rabbits, whereas the breadth (width) of the skull remained similar in the different sized domestic rabbits, despite differences in overall body size (Böhmer & Böhmer, 2017; Darwin, 1875; Hückinghaus, 1965a, 1965b; Klatt, 1913). These differences of relative cranial dimensions between wild and domestic rabbits were not attributable to size differences alone, but found to represent “true” proportional changes due to domestication (Böhmer & Böhmer, 2017; Hückinghaus, 1965a, 1965b). Darwin (1875) argued that the relatively elongated skull in domestic rabbits—particularly concerning the facial portion—actually results from a reduction of brain size due to domestication, which has repeatedly been reported in rabbits (Brusini et al., 2018; Choinowski, 1958; Darwin, 1875; Fischer, 1973; Hückinghaus, 1965a, 1965b; Klatt, 1912; Müller, 1919; for a review, see Balcarcel, Geiger, et al., 2021). Our findings are in accordance with these previous studies, suggesting that the facial portion of the skull appears relatively long in domestic rabbits due to the smaller braincase dimensions, but is actually not shorter in absolute terms (Figure 1). Although there is thus no evidence for the “domestication syndrome” regarding facial size, the repeatedly reported braincase reduction across various rabbit varieties is in accordance with the hypothesis (e.g., Herre & Röhrs, 1990). The independence of cranial differences from size differences in wild versus domestic rabbits might be attributable to different ontogenetic growth trajectories between the two (Sánchez‐Villagra et al., 2017). In general, morphological differences in domestic versus wild rabbits might also be due to phenotypic plasticity and widespread feeding practices in domestic rabbit keeping and breeding, which are different from the dietary habits of wild rabbits (Böhmer & Böhmer, 2017).

Further contributing to the flat appearance of the skull in domestic rabbits is the size‐independent, less klinorhynchic configuration of the face and the cranium, that is, the rostrum has been found to be less ventrally angled in domestic compared to wild rabbits (Hückinghaus, 1965a, 1965b). In the latter, there is a pronounced flexure near the basisphenoid/presphenoid juncture (Kraatz et al., 2015). Also here, our findings are in accordance with these previous characterizations (Figure 1). Among wild leporid species, it has been found that klinorhynchy is more pronounced in cursors and saltators compared to generalists, probably due to the need for an expanded visual field in the first (Kraatz & Sherratt, 2016; Kraatz et al., 2015). This suggests that domestication might has led to a release from selective pressures—notably predation—that in the evolutionary past of this lineage have led to the evolution towards a saltatorial mode of locomotion and a pronounced facial tilt.

Besides these characteristics, Darwin (1875) also pointed out a number of more detailed cranial modifications that appear to be related to domestication and breed formation in rabbits, including the relative size and shape (and orientation) of the auditory meatus, the interparietal bone, the supraorbital plate, and the foramen magnum. These characteristics were not investigated here. However, we characterized some additional cranial characteristics associated with rabbit domestication. In particular, we found that cheek‐tooth row length was reduced in the domestic sample compared with the wild one, which is in accordance with the “domestication syndrome” (Figure 1), but not in accordance with a previous study finding that there was no change in tooth row length between wild and domestic rabbits (Böhmer & Böhmer, 2017). In this study, skull shape of 12 “wild” rabbits from Germany and Austria and 12 domestic rabbits of no specific breed (all mature) was quantified on radiographs using a two‐dimensional approach (lateral view) (Böhmer & Böhmer, 2017). The discrepancy between this and our study concerning tooth row length differences in wild versus domestic rabbits might be a result of a different methodology (two‐ vs. three‐dimensional) and/or sample and point to a need for further studies. Although we did not quantify the size of the individual teeth, this reduction of the length of the entire tooth row is indicative of a general decrease in tooth size in the domestic sample. Further, there appears to be a very slight broadening of the snout in the domestic sample, also in accordance with the “domestic syndrome” (Figures 1).

We further tested whether neural crest‐derived parts of the skull are more variable compared with the mesoderm‐derived parts, in line with the “neural crest hypothesis” of the “domestication syndrome” (Wilkins et al., 2014). We found support for this hypothesis, where domestic rabbits showed almost twice as much variation in the module of landmarks related to the neural crest (NC) region (Figure 5).

### 3.2 Cranial shape changes within the domestic rabbit sample

Archaeological remains of domestic rabbits were only distinguishable from their wild counterparts form the 18th century onwards (Irving‐Pease et al., 2018 and references therein). Comparing dwarf, medium‐sized, and giant domestic breeds, it was found that adult dwarf rabbits had relatively shorter and broader faces compared to the medium‐sized ones, thus resembling juvenile stages of the latter (Fiorello & German, 1997). No such differences were found between medium‐sized and giant rabbits (Fiorello & German, 1997). Further, the ventral angulation of the premaxilla versus the palate (klinorhynchy) among domestic rabbits was found to be loosely attributable to body size, with large breeds showing a more pronounced klinorhynchy and therefore a wider distance between the anterior tips of the nasals and the palate than smaller breeds, that is, a higher anterior rostrum (Hückinghaus, 1965a, 1965b). In our sample, we found that among the domestic rabbits, the braincase dimensions were disproportionally large in small rabbits, whereas facial length scaled proportionally with body size. This pattern was particularly conspicuous in the smallest of the investigated breeds, the “Hermelin” rabbits, which clustered on the far left of the regression plot (Figure 3). This scaling pattern would contribute to cranial proportional changes similar to the one described previously (Fiorello & German, 1997).

Although these scaling relationships lead to relatively short faces in small domestic rabbits (and thus to a pattern which might be described as “allometric” brachycephaly; Geiger et al., 2021), this appears to be purely the result of negatively allometric scaling of the braincase. Thus, although the here reported scaling relationship of braincase size among domestic rabbits is in accordance with the “rule of Haller” (e.g., Bauchot, 1978; Bronson, 1979; Emerson & Bramble, 1993; Gould, 1975; Klatt, 1913; Lüps, 2008; Radinsky, 1985), the scaling relationship of the face does not corroborate with the “cranial evolutionary allometry hypothesis” (CREA), which suggest that larger forms of the same species or larger species within a clade have relatively long faces (positive allometry; e.g., Cardini & Polly, 2013), and which has been substantiated in a number of different taxa (Bright et al., 2016; Cardini, 2019; Cardini & Polly, 2013; Cardini et al., 2015; Emerson & Bramble, 1993; Le Verger et al., 2020; Radinsky, 1985; Tamagnini et al., 2017). Exceptions from CREA have previously also been reported for African bovids and equids (Cardini, 2019) and among domestic horses (Clauss et al., 2022; Heck et al., 2019). These cases constitute examples of the “long face hypothesis,” suggesting that small grazers have relatively long faces, probably due to functional requirements related to energy intake and tooth size (Clauss et al., 2022; Heck et al., 2019; Spencer, 1995). Relatedly, our results suggest that tooth row length in rabbits is disproportionally large in small varieties, also in accordance with findings from other mammals and possibly caused by a slower evolutionary rate of dental change relative to the skeleton (Clauss et al., 2022).

## 4 MATERIALS AND METHODS

### 4.1 Specimens

For this study, we used crania from 30 European rabbits (*O. cuniculus* Linnaeus, 1758), from which 16 were wild and 14 domestic (Supporting Information). Specimens are housed at the Zoologisches Institut/Populationsgenetik (former Institut für Haustierkunde), Christian‐Albrechts‐Universität zu Kiel, Germany (I.f.H.), and the Senckenberg Museum Frankfurt am Main, Germany. There were no experiments conducted in any living animal, nor were any animals killed for this study.

Specimens were categorized as “wild” or “domestic” depending on information obtained from collection databases and labels. Wild rabbits were here defined as the ones that were not specifically designated as domesticates and which have been sampled in the wild and in regions that are part of the early Holocene distribution area of the species (Kurtén, 1986), before their human induced spread throughout Europe (Ferrand, 2008 and references therein; Carneiro et al., 2011; Irving‐Pease et al., 2018; Lopez‐Martinez, 2008). Specifically, these were localities on the Iberian Peninsula (Spain and Portugal) and the South‐West of France (St. Bonnet du Gard). Although these presumably wild rabbits might constitute feral populations of domestic rabbits or have been subject to introgressive hybridization with feral domestic populations, similar approaches proved to be adequate in genetic studies (Carneiro et al., 2011). Domestic rabbits were here defined as the ones which were clearly marked as such in the collection database (e.g., as *O. cuniculus* f. domesticus) and/or if they were donated to the respective collection by rabbit breeders.

Although wild European rabbits’ body mass ranges between about 1–1.7 kg (Kaetzke et al., 1984), the body mass range of domestic rabbits is much greater and spans 1–9 kg (Bökönyi, 1974; Robinson, 1984). Our sample contains various different domestic rabbit breeds (“Klein Chinchilla,” “Hermelin,” “Schwarzloh,” “Holländer,” “Russe,” and “Angora”) and specimens from which carcass weight is known from collection labels span a body mass range of 1–4.5 kg. Thus, our sample covers a large part of the body mass range of domestic rabbits. Although our sample is limited in that it contains only modern breeds, rather than archaeological specimens representing the first stages in the domestication process, a shared character among modern breeds could be indicative of the presence of that character in all breeds, thus being a hallmark of domestication rather than breed formation (Lord et al., 2020).

Both sexes were used in this study. Only skeletally and dentally mature specimens were used to account for effects of ontogenetic variation on cranial shape. Dental maturity was defined as the presence of the third molar in the upper and lower jaw, which is the last permanent tooth to erupt in rabbits (Habermehl, 1975). Skeletal maturity was defined as the occipital bone being fused to the rest of the cranium (Habermehl, 1985) (the occipital bone is usually detached in dry crania of juvenile rabbits). Only specimens exhibiting no craniodental pathologies were used.

### 4.2 Computed tomography (CT) scans and landmarks

All crania were scanned using a high‐resolution X‐ray micro‐CT scanner (XT H 225 ST, Nikon Metrology) at the Palaeontological Institute and Museum at the University of Zurich, Switzerland. Three‐dimensional surface models of the crania were generated in Avizo® 2020.2.0 (FEI Visualization Science Group). Landmarks were placed on the resulting 3D surface reconstructions using MeshLab version 2021.07.

Fourteen landmarks were digitized on one‐half of the 3D surface models and chosen to represent major morphofunctional parts of the cranium (Figure 2 and Table 1). These were adapted from Balcarcel, Sánchez‐Villagra, et al. (2021), Ge et al. (2015), Geiger et al. (2017), Heck et al. (2018), and Wilson et al. (2021), with anatomical descriptions based also on Wible (2007). Information on specimens and raw landmark data are provided in the Supporting Information data.

**Table 1:** Landmarks used in this study

No.	Developmental module	Description
1	NC	Point where premaxillary bone and incisor meet at the groove on the buccal side of the tooth
2	NC	Junction of premaxilla, nasal, and frontal bones
3	NC	Anterior‐most point of the zygomatic process, measured on the tip of the process
4	NC	Nasion, nasal‐frontal suture, midline
5	NC	Posterior‐most point of the jugal‐squamosal suture
6	NC	Bregma, intersection of interfrontal and interparietal sutures
7	MD	Inion, highest projection of the external occipital protuberance (dorsal), at the midline
8	NC	Anterior‐most tip of the second premolar alveolus (i.e., first cheek tooth)
9	NC	Posterior‐most tip of the third molar alveolus (i.e., last cheek tooth)
10	NC	Anterior point of the incisive and palatal foramen along longitudinal axis of cranium
11	MD	Anterior rim of basioccipital bone, midline
12	MD	Posterior‐most point of rim of carotid canal
13	MD	Basion, ventral margin of the foramen magnum (central point of the torus/buldge)
14	MD	Opisthion, dorsal margin of the foramen magnum

*Note*: No. refers to the landmark number indicated in Figure 2. Developmental module refers to the NC or MD origins of the bones where these landmarks were set according to Mishina and Snider (2014).

Abbreviations: MD, mesoderm; NC, neural crest.

For the analysis of cranial module shape disparity, each landmark was categorized by tissue of origin (i.e., neural crest [NC] or mesoderm [MD] derived) of the bone on which that landmark was located, following Mishina and Snider (2014) (Figure 2 and Table 1). Landmark No. 5 (Bregma), which was located on the boundary between the NC and MD portions of the cranium (Figure 2 and Table 1), was assigned to the MD module based on the mesoderm origin of the coronal suture (Mishina & Snider, 2014; Wilson et al., 2021).

### 4.3 Analyses

We performed a generalized Procrustes superimposition to remove the effects of scale, orientation, and position from the landmark data set using the “gpagen a” function in *geomorph* R package (Adams et al., 2022). Then, we computed a PCA based on a covariance matrix (using “gm.prcomp” in *geomorph*) to examine shape variation across the wild and the domestic groups. This analysis is hereafter termed “allometry‐included analysis.”

To visualize the relationship between cranial shape and size we regressed the shape variables against log‐transformed centroid size and computed the shape score (regression score), which is a univariate summary of the regression fit (Drake & Klingenberg, 2008). To test whether the wild and the domestic groups had a different allometric trajectory, we used an ANCOVA model with an interaction term of size:group. These analyses were implemented with the “procD.lm” function in *geomorph*. Then, to remove the allometric component of shape, we extracted the regression residuals, and performed a second PCA. This analysis is hereafter termed “allometry‐adjusted analysis.”

To test for shape differences between the wild and the domestic groups, we performed Procrustes ANOVA on both allometry‐included and allometry‐adjusted data, using the “procD.lm” function in *geomorph*. Then, we performed morphological disparity analyses to calculate the amount of shape variation in each group on each data set. Statistical significance was assessed through permutations (1000 iterations), implemented with the “morphol.disparity” function in *geomorph*.

To analyze cranial module shape disparity, we subdivided the non‐allometry‐adjusted and allometry‐adjusted data sets into the NC and MD portions (see above and Figure 2). Then, we computed the Procrustes variance of each group, divided by the number of landmarks in each module to standardize the variance and test whether the variance of the NC portion of the cranium was greater in the domestic sample compared with the wild. Statistical significance was assessed through permutations (1000 iterations), implemented with the “morphol.disparity” function in *geomorph*.

To test specific predictions in conjunction with the “domestication syndrome” hypothesis, at least as concerned with a survey of current, diverse rabbit breeds, we extracted linear dimensions from the landmark data using the Pythagoras theorem implemented with “interlmkdist” function in *geomorph* (Table 2). All extracted linear dimensions were then log transformed. The logarithmized cranial base length was subsequently used as a proxy for body size (Lüps, 1974) and all other log‐linear dimensions were divided by this proxy to obtain size‐independent indices. We then used nonparametric Wilcoxon–Mann–Whitney tests to compare these indices between the wild and the domestic group, implemented with “wilcox.test” function in R package *stats* (R Core Team, 2022).

**Table 2:** Linear dimensions extracted from landmarks

Linear dimensions	Landmarks
Cranial base length (body size proxy)	11 and 13
Facial length	1 and 2
Braincase height	6 and 13
Braincase length	6 and 7
Braincase width	6 and 5
Tooth row length	8 and 9

*Note*: The landmarks that have been used to define the linear dimensions are corresponding to the ones in Table 1 and Figure 2.

To further scrutinize underlying patterns of skull dimension changes in rabbit domestication and breed formation, we analyzed scaling relationships of the extracted, log‐transformed cranial dimensions (Table 2) with body size among the domestic rabbits. For this, we performed ordinary least‐squares linear regressions with the body size proxy as the independent and the cranial dimensions as the dependent variable. Slopes were interpreted as “isometric” if the 95% CI included 1 and as “negatively allometric” or “positively allometric” if the 95%" CI was below or above 1, respectively (see also Clauss et al., 2022) for detailed description and rationale of the approach). All analyses were performed using R version 4.2.0 (R Core Team, 2022). The R‐script is available via the online supplement.

## 5 CONCLUSIONS

Our preliminary results of skull shape variation among a broad but not comprehensive sample of rabbit breeds shows an expansion of the morphospace in comparison to the wild form. Our results and previous studies, suggest that some, but not all of the predicted characteristics of the “domestication syndrome” are present in rabbits. Although these findings do not per se refute the validity of the “domestication syndrome,” they add to the growing body of literature, suggesting more complex processes behind the patterns that we see in domestication. Future studies would benefit from greater sampling of different varieties, including giant breeds and “half‐lops” (Darwin, 1875) and wild individuals from world‐wide localities where *Oryctolagus* have been introduced.

## AUTHOR CONTRIBUTIONS

Madeleine Geiger and Marcelo R. Sánchez‐Villagra conceptualized the study. Madeleine Geiger collected the data, reviewed the literature, and wrote the manuscript. Emma Sherratt and Madeleine Geiger performed the analyses. Marcelo R. Sánchez‐Villagra and Emma Sherratt critically revised the manuscript. All authors gave final approval for publication.

## CONFLICT OF INTEREST

The authors declare no conflict of interest.

### PEER REVIEW

The peer review history for this article is available at https://publons.com/publon/10.1002/jez.b.23171

## Supporting information

Supporting information.

Supporting information.

## Data Availability

The supplementary data file provides all raw data used in this study.
