# Supplementary material for: Cranial shape variation in domestication: A pilot study on the case of rabbits
Source: J Exp Zool B Mol Dev Evol. 2022 Aug 7;338(8):532–41. doi: 10.1002/jez.b.23171 (PMC9804214; doi:10.1002/jez.b.23171)
Supplement: Supplementary file 2 — Supporting information. [file JEZ-338-532-s002.docx]

**Supplementary Information**

**
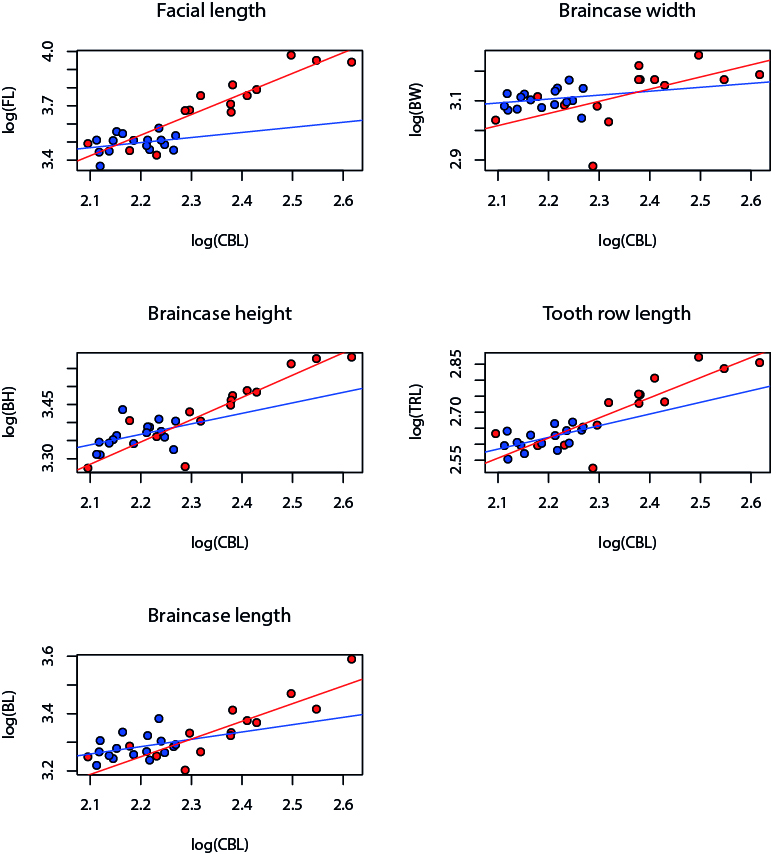
**

**Supplementary Figure S1.** Ordinary least-squares regressions of the extracted, log-transformed cranial dimensions with body size (Cranial base length as body size proxy, CBL).

**R-Script used for this study**

# Analyses for Geiger, Sánchez-Villagra & Sherratt

# "Cranial shape variation in domestication: a pilot study on the case of rabbits"

# Journal of Experimental Zoology, part B.

# May 2022

# Code by Emma Sherratt

# R version 4.2.0

library(geomorph) # v.4.0.3

library(readxl) # v.1.4.0

# save .xls as .csv first. Then set WD to folder with file

rawdata <- read_excel("MS_RabbitMorphometry_SupplementaryData.xlsx", sheet =1, col_names = TRUE,na = "na")

Y <- arrayspecs(rawdata[,c(10:51)], k=3, p=14) # extract coordinate data

dimnames(Y)[[3]] <- rawdata$Collection_ID # add collection_ID to coordinates array

W <- which(rawdata$Form=="wild")

D <- which(rawdata$Form=="domestic")

rawdata$Form <- factor(rawdata$Form) # make as factor

# Create a colour factor for the groups:

colours <- c("red", "blue")

names(colours) <- levels(rawdata$Form)

col <- colours[match(rawdata$Form, names(colours))]

GPA <- gpagen(Y) # Procrustes superimposition

plotAllSpecimens(GPA$coords) # look at superimposed data

gdf <- geomorph.data.frame(GPA, form = rawdata$Form) # make as geomorph dataframe for further analyses

# Principal components analysis

PCA <- gm.prcomp(GPA$coords) # Principal components analysis

# Wireframe for plotting

wireframe <- matrix(c(1,4,4,6,1,10,4,2,10,8,8,9,2,3,3,9,3,5,5,6,6,7,7,14,12,14,12,13,11,13,9,11), ncol=2, byrow = TRUE)

#### Non-allometry adjusted analyses ####

# Figure 2 Non-allometry adjusted PCA

PCA.plot <- plot(PCA, pch=21, bg=col, asp=TRUE, cex=GPA$Csize/50) # plot PC axes 1 vs 2, coloured by "Form" grouping, point size by Csize

shapeHulls(PCA.plot,groups=rawdata$Form, group.cols = colours) # hulls

legend(x=0.02, y=0.05, legend = levels(rawdata$Form), col=colours, pch=19, bty="n") # legend

text(PCA$x[D,1:2], labels = rawdata$Breed[1:14], pos=4, cex=0.8) # add breed names

# Wireframes of min and max PC1.

layout3d(mat=matrix(c(1:4), ncol=2, byrow = TRUE), sharedMouse = FALSE)

plotRefToTarget(GPA$consensus, PCA$shapes$shapes.comp1$min, method = "points", links = wireframe) # mean to min, dorsal

plotRefToTarget(GPA$consensus, PCA$shapes$shapes.comp1$max, method = "points", links = wireframe) # mean to max, dorsal

plotRefToTarget(GPA$consensus, PCA$shapes$shapes.comp1$min, method = "points", links = wireframe) # mean to min, lateral

plotRefToTarget(GPA$consensus, PCA$shapes$shapes.comp1$max, method = "points", links = wireframe) # mean to max, lateral

# Rotate int position then save image using RGL snapshot

rgl.snapshot("Figure 2B.png")

# Test for disparity between groups

MD.res <- morphol.disparity(f1=coords ~ 1, groups = ~form, data = gdf)

# Procrustes variances for defined groups

# domestic wild

# 0.003516133 0.002105080

# P-Values

# domestic wild

# domestic 1.000 0.008

# wild 0.008 1.000

# Multivariate regression to test for allometry

fit <- procD.lm(f1=coords ~ log(Csize), data = gdf)

summary(fit)

# Df SS MS Rsq F Z Pr(>F)

# log(Csize) 1 0.021723 0.0217231 0.26202 9.9412 3.3472 0.001 **

# Residuals 28 0.061184 0.0021851 0.73798

# Total 29 0.082907

# 26% of shape variation due to size

# An ANCOVA model to test for different allometric slopes between groups

fit1 <- procD.lm(f1=coords ~ log(Csize)*form, data = gdf) # Multivariate regression

summary(fit1)

# Df SS MS Rsq F Z Pr(>F)

# log(Csize) 1 0.021723 0.0217231 0.26202 13.621 3.6709 0.001 **

# form 1 0.018815 0.0188150 0.22694 11.798 4.1504 0.001 **

# log(Csize):form 1 0.000904 0.0009042 0.01091 0.567 -1.0509 0.843

# Residuals 26 0.041465 0.0015948 0.50014

# Total 29 0.082907

# Interaction term NS, so not significantly different slopes

# group term sig so different intercepts

# Given the NS interaction term, most appropriate model uses + not *:

fit1 <- procD.lm(f1=coords ~ log(Csize)+form, data = gdf)

plot(fit1, type = "regression",

predictor = log(gdf$Csize), reg.type = "RegScore",

pch = 21, bg = col) # Regression score; plots the common allometric component of the two groups (i.e. overlapped)

predlineplot <- plot(fit1, type = "regression",

predictor = log(gdf$Csize), reg.type = "PredLine",

pch = 21, bg = col) # Predicted plot, sensu Adams & Nistri 2010

# Figure 3

regscore.plot <- plot(fit, type = "regression",

predictor = log(gdf$Csize), reg.type = "RegScore",

pch = 21, bg = col, cex=gdf$Csize/50, xlab="log-centroid size")

# overal predicted line

points(y=predlineplot$PredLine*-1, x=log(gdf$Csize), pch = 19, cex=0.6)

text(x=log(gdf$Csize)[1:14], y=regscore.plot$RegScore[1:14], labels = rawdata$Breed[1:14], pos=4, cex=0.8) # add breed names

legend(x=4.7, y=0, legend = levels(rawdata$Form), col=colours, pch=19, bty="n")

# Procrustes ANOVA to test for differences in shape between groups

fit2 <- procD.lm(f1=coords ~ form, data = gdf)

summary(fit2)

# Df SS MS Rsq F Z Pr(>F)

# form 1 0.032790 0.03279 0.3955 18.319 4.0366 0.001 **

# Residuals 28 0.050117 0.00179 0.6045

# Total 29 0.082907

#### Allometry-adjusted analyses ####

# Take residuals from fit1 above

Y.resid <- fit1$GM$residuals + array(GPA$consensus, dim(PCA$A)) # add consensus shape to residuals and transform into 3D array

PCA.allofree <- gm.prcomp(Y.resid)

# Figure 4 Non-allometry adjusted PCA

PCA.plot2 <- plot(PCA.allofree, pch=21, bg=col, asp=TRUE, cex=GPA$Csize/50) # plot PC axes 1 vs 2, coloured by "Form" grouping, point size by Csize

shapeHulls(PCA.plot2,groups=rawdata$Form, group.cols = colours) # hulls

legend(x=-0.06, y=-0.02, legend = levels(rawdata$Form), col=colours, pch=19, bty="n") # legend

MDallofree.res <- morphol.disparity(f1=Y.resid ~ 1, groups = rawdata$Form)

# Procrustes variances for defined groups

# domestic wild

# 0.001744995 0.001121199

# P-Values

# domestic wild

# domestic 1.00 0.04

# wild 0.04 1.00

#### Module Analyses ####

MD <- c(7,11:14) # Mesoderm

NC <- c(1:6,8:10) # Neural Crest

PCA.MD <- gm.prcomp(GPA$coords[MD,,])

PCA.NC <- gm.prcomp(GPA$coords[NC,,])

layout(c(1,2))

PCA.MD.plot <- plot(PCA.MD, pch=21, bg=col, asp=TRUE, cex=GPA$Csize/50)

title("Mesoderm")

shapeHulls(PCA.MD.plot,groups=rawdata$Form, group.cols = colours) # hulls

PCA.NC.plot <- plot(PCA.NC, pch=21, bg=col, asp=TRUE, cex=GPA$Csize/50)

title("Neural Crest")

shapeHulls(PCA.NC.plot,groups=rawdata$Form, group.cols = colours) # hulls

layout(1)

# Procrustes variance (disparity) per module, taking into account number of lmks in each module

MD.gp <- morphol.disparity(f1=coords[MD,,] ~ 1, groups = rawdata$Form, data = gdf)

# Procrustes variances for defined groups

# domestic wild

# 0.0006768077 0.0004883428

# P-Values

# domestic wild

# domestic 1.000 0.094

# wild 0.094 1.000

NC.gp <- morphol.disparity(f1=coords[NC,,] ~ 1, groups = rawdata$Form, data = gdf)

# Procrustes variances for defined groups

# domestic wild

# 0.002839326 0.001616737

#

# P-Values

# domestic wild

# domestic 1.000 0.008

# wild 0.008 1.000

# Figure 5

barplot(c(MD.gp$Procrustes.var/length(MD),NC.gp$Procrustes.var/length(NC)),

col=c(colours,adjustcolor(colours, alpha=0.4)), ylab = "Procrustes variance",

xlab="Mesoderm vs. Neural Crest")

#### Linear dimension analyses ####

CBL <- interlmkdist(A= Y, lmks = c(11,13)) #Cranial base length (body size proxy)

FL <- interlmkdist(A= Y, lmks = c(1,2)) #Facial length

BH <- interlmkdist(A= Y, lmks = c(6,13)) #Braincase height

BL <- interlmkdist(A= Y, lmks = c(6,7)) #Braincase length

BW <- interlmkdist(A= Y, lmks = c(6,5)) #Braincase width

TRL <- interlmkdist(A= Y, lmks = c(8,9)) #Tooth row length

# Mann-Whitney-Wilcoxon Test

wilcox.test((log(FL)/log(CBL)) ~ rawdata$Form)

# W = 84, p-value = 0.2572

wilcox.test((log(BH)/log(CBL)) ~ rawdata$Form)

# W = 27, p-value = 0.0001847

wilcox.test((log(BL)/log(CBL)) ~ rawdata$Form)

# W = 28, p-value = 0.000228

wilcox.test((log(BW)/log(CBL)) ~ rawdata$Form)

# W = 24, p-value = 9.536e-05

wilcox.test((log(TRL)/log(CBL)) ~ rawdata$Form)

# W = 32, p-value = 0.000506

# Ordinary least-squares regressions

# Supplementary figure

layout(matrix(c(1:6), ncol=2))

plot(x=log(CBL), y=log(FL), pch=21, bg=col, main = "Facial length")

fit.FL.W <- lm(log(FL[W]) ~ log(CBL[W]))

abline(fit.FL.W, col="blue")

fit.FL.D <- lm(log(FL[D]) ~ log(CBL[D]))

abline(fit.FL.D, col="red")

plot(x=log(CBL), y=log(BH), pch=21, bg=col, main = "Braincase height")

fit.BH.W <- lm(log(BH[W]) ~ log(CBL[W]))

abline(fit.BH.W, col="blue")

fit.BH.D <- lm(log(BH[D]) ~ log(CBL[D]))

abline(fit.BH.D, col="red")

plot(x=log(CBL), y=log(BL), pch=21, bg=col, main = "Braincase length")

fit.BL.W <- lm(log(BL[W]) ~ log(CBL[W]))

abline(fit.BL.W, col="blue")

fit.BL.D <- lm(log(BL[D]) ~ log(CBL[D]))

abline(fit.BL.D, col="red")

plot(x=log(CBL), y=log(BW), pch=21, bg=col, main = "Braincase width")

fit.BW.W <- lm(log(BW[W]) ~ log(CBL[W]))

abline(fit.BW.W, col="blue")

fit.BW.D <- lm(log(BW[D]) ~ log(CBL[D]))

abline(fit.BW.D, col="red")

plot(x=log(CBL), y=log(TRL), pch=21, bg=col, main = "Tooth row length")

fit.TRL.W <- lm(log(TRL[W]) ~ log(CBL[W]))

abline(fit.TRL.W, col="blue")

fit.TRL.D <- lm(log(TRL[D]) ~ log(CBL[D]))

abline(fit.TRL.D, col="red")

# Extract slope estimates and 95% CI

summary(fit.FL.D)

confint(fit.FL.D, level=0.95)

summary(fit.BH.D)

confint(fit.BH.D, level=0.95)

summary(fit.BL.D)

confint(fit.BL.D, level=0.95)

summary(fit.BW.D)

confint(fit.BW.D, level=0.95)

summary(fit.TRL.D)

confint(fit.TRL.D, level=0.95)
